# Remarks on the Proper Mode of Administering Sulphuric Ether by Inhalation

**Published:** 1847-10

**Authors:** 


*Remarks on the Proper Mode of Administering Sulphuric Ether by Inhalation.* By William T. G. Morton; 12mo, pp. 44, Boston, 1847.

The above work, dedicated to the Surgeons of the Massachusetts General Hospital, is beautifully printed. It is designed to serve as a guide in the administration by inhalation of sulphuric ether for the relief of pain during surgical operations, and to such especially as are inexperienced in the use of this most subtle and powerful agent, it will be found highly valuable. The directions which it contains upon the subject are clearly expressed, and we doubt not, if they were always followed, its *liability* to produce dangerous effects would be very greatly lessened.

With regard to the efficiency of this agent, when inhaled, in the prevention of pain in surgical operations, there can be no question; this fact has been well established, and we believe too, it may be taken in a large majority of the cases with comparative impunity; but still, we believe it should only be administered for the prevention of pain in very severe and protracted surgical operations. As for ourself, though we extract a considerable number of teeth every day, we do not meet with half a dozen cases a year in which we have any difficulty in persuading our patients to submit to the operation; and thus far, we have succeeded in prevailing on nearly all of the subjects of even these, though not so easily, to submit to it. For so simple an operation as that of the extraction of a tooth, we do not think the inhalation of the vapor of ether should be resorted to, or, at any rate, except in some *very rare* cases. Certainly, we have not met with a case, since the discovery of the application of this agent for the prevention of pain, in which we should have felt ourself justifiable in using it.

The discovery, however, may prove valuable, and Dr. Morton is evidently entitled to the honor and credit of the principal authorship of it. The abandonment of the patent which he had secured for its use, is also creditable to him in the highest degree. But our own views with regard to the propriety of using this agent are so ably and fully expressed in another part of the present number of the Journal, by our colleague, Dr. Dwinelle, it is unnecessary for us to say more upon the subject at present. We ask for this article a careful and attentive perusal.—*Balt. Ed.*

